# Supplementary material for: Socioeconomic Inequalities and Ethnicity Are Associated with a Positive COVID-19 Test among Cancer Patients in the UK Biobank Cohort
Source: Cancers (Basel). 2021 Mar 25;13(7):1514. doi: 10.3390/cancers13071514 (PMC8037247; doi:10.3390/cancers13071514)
Supplement: Supplementary file 1 [file cancers-13-01514-s001.pdf]

SUPPLEMENTARY MATERIALS

Files in this data supplement:

Supplementary Table S1. Distribution of Incident Cancer subtypes (ICD-10 code) for the Case-Cohort study at Baseline in 16th March, 2020 (N = 18,917)

Supplementary Table S2. Sensitivity analyses based on two modified versions of the control population: scenario 1 where the controls were only those patients with a negative test result and scenario 2 where controls were only those patients non-tested for COVID-19, excluding those with a negative result.

Supplementary Technical notes 1. Sensitivity analyses

Supplementary Figure S1. Case-cohort study with case-base design

Supplementary Table S1. Distribution of Incident Cancer subtypes (ICD-10 code) for the Case-Cohort study at Baseline in 16th March, 2020 (N = 18,917)

Cancer Sites	Cases, n (%)	All cohort N
**Melanoma**	3 (0.2)	1,336
**Lip, oral cavity, and pharynx**	1 (0.3)	382
**Digestive organs (non-colorectal)**	3 (0.7)	415
**Colorectal**	10 (0.5)	2,149
**Breast**	21 (0.4)	4,955
**Lung**	4 (1.0)	386
**Prostate**	21 (0.4)	5,020
**Central nervous system**	1 (1.0)	96
**Mesothelial and soft tissue**	1 (0.7)	139
**Urogenital cancer**	10 (0.5)	2,087
**Respiratory and intrathoracic organs**	0 (0.0)	93
**Bone & articular cartilage**	0 (0.0)	24
**Endocrine glands**	2 (0.9)	229
**Hematological cancer**	12 (0.8)	1,518
**Other neoplastic conditions**	0 (0.0)	88
**Total**	89 (0.5)	18,917

Abbreviation: ICD-10, International Classification of Diseases, Tenth Revision

Supplementary Table S2. Sensitivity analyses based on two modified versions of the control population: scenario 1 where the controls were only those patients with a negative test result and scenario 2 where controls were only those patients non-tested for COVID-19, excluding those with a negative result

Variables	*Scenario 1<br>aRR (95% CI)	*Scenario 2<br>aRR (95% CI)
**Townsend Deprivation Index**		
Quintiles 2nd vs. 1st	2.09 (0.86–5.11)	2.08 (0.84–5.12)
Quintiles 3rd vs. 1st	1.96 (0.79–4.87)	2.02 (0.81–5.05)
Quintiles 4th vs. 1st	1.46 (0.60–3.56)	1.79 (0.72–4.45)
Quintiles 5th vs. 1st	1.88 (0.76–4.62)	2.58 (1.03–6.50)
**Sex** male vs. female	0.93 (0.57–1.52)	1.22 (0.73–2.06)
**Age** per ten-year increase	0.91 (0.57–1.46)	1.12 (0.67–1.87)
**Ethnicity**		
Asian vs. White	1.56 (0.20–11.80)	1.13 (0.14–9.22)
Black vs. White	6.72 (2.84–15.88)	5.67 (1.82–17.65)
Others vs. White	2.85 (0.83–9.77)	2.81 (0.7 3–10.89)
**Employment status**		
Retired vs. employed	1.39 (0.79–2.46)	1.39 (0.79–2.46)

Unemployed/unpaid vs. employed	2.35 (1.06–5.20)	2.45 (1.10–5.44)
**Smoking status**		
Current smoker vs. non-smoker	1.20 (0.51–2.85)	1.30 (0.53–3.16)
Ex-smoker vs. non-smoker	1.28 (0.77–2.12)	1.56 (0.95–2.56)
**BMI**		
Per 5 kg/m^2 increase	1.22 (1.03–1.44)	1.44 (1.21–1.70)
**Malignancy type**		
Haematological vs. melanoma and others	2.61 (0.57–12.01)	4.06 (0.87–18.97)
Non-haematological vs. melanoma and others	2.49 (0.62–10.02)	2.29 (0.56–9.44)
**Years of cancer diagnosis**		
Within 5 years vs. beyond 5 years of diagnosis	1.31 (0.76–2.25)	1.45 (0.82–2.58)
**Marital status**		
With a partner vs. without a partner	2.17 (0.64–7.36)	2.38 (0.67–8.48)

Abbreviations: aRR, adjusted risk ratio; BMI, body mass index

*Adjusted for Townsend Deprivation Index, age, and sex, ethnicity, employment status, smoking status. BMI in kg/m^2, malignancy type, years of cancer diagnosis, marital status

Supplementary Technical notes 1. Sensitivity analyses

We explored different modelling specifications, including changing the functional form of continuous covariates and the interactions between the deprivation and BMI, sex, ethnicity, smoking, and employment status. We also explored the association between deprivation and COVID-19 for subgroup analysis based on the absolute number of cases by cancer site (i.e., more than 20 COVID-19 positive cases) for breast and prostate cancers. We explored the consistency of the case-base design and assumptions using two modified versions of the control population: i) where the controls were only those patients with a negative test result and ii) where controls were only those patients non-tested for COVID-19, excluding those with a negative result. To assess the consistency of our results for socioeconomic deprivation based on a complete-case analysis against the completely-at-random assumption for the missing data, we developed a strategy using a multiple imputation by chained equations. We imputed 50 datasets for the variables ethnicity, smoking, employment and marital status using logistic, multinomial and linear conditional regression models and results were combined using Rubin's rules [1].

In the sensitivity analysis, we found that multivariate adjusted models were consistent to different modelling specifications, functional forms of continuous covariates and the modified case-base sampling design strategy. There was no effect modification of deprivation by levels of BMI, sex, ethnicity, smoking, and employment status. Finally, results after multiple imputation were consistent with our results for socioeconomic deprivation based on a complete-case analysis (Model 9, Table 2).

Reference:

1. Rubin, D.B. Inference and missing data. *Biometrika* **1976**, *63*, 581-592, doi:10.1093/biomet/63.3.581.

Supplementary Figure S1. Case-cohort study with case-base design

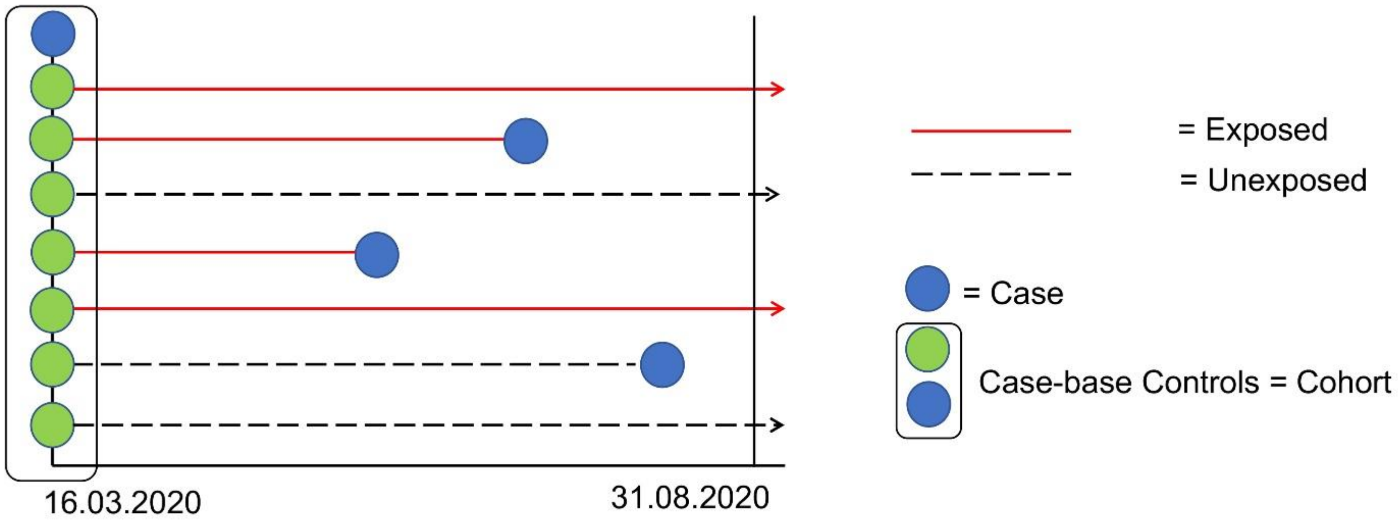

	Cases	Controls
Exposed	A_1	$N_1 * f_1$
Unexposed	A_0	$N_0 * f_0$

f = proportion of non-cases selected as controls

If $f_1 = f_0$, then

$$\text{Odds ratio} = \frac{A_1/N_1 * f_1}{A_0/N_0 * f_0} = \frac{A_1/N_1}{A_0/N_0}$$

The result is a risk ratio (RR)
